# Separating Acute Rheumatic Fever from Nonrheumatic Streptococcal Myocarditis

**DOI:** 10.1155/2019/4674875

**Published:** 2019-01-16

**Authors:** Laith A. Derbas, Anweshan Samanta, Srinivasa Potla, Moustafa Younis, Laura M. Schmidt, Ibrahim M. Saeed

**Affiliations:** ^1^Department of Internal Medicine, University of Missouri-Kansas City, Kansas City, MO, USA; ^2^Mid America Heart Institute, Saint Luke's Hospital, Kansas City, MO, USA

## Abstract

**Introduction:**

Streptococcal pharyngitis has been historically complicated with systemic involvement manifesting as acute rheumatic fever, which is a serious condition that can lead to permanent damage to heart valves. A recent association between streptococcal pharyngitis and nonrheumatic heart disease is emerging in literature. We present a case of nonrheumatic streptococcal myocarditis diagnosed using cardiac MRI.

**Case Presentation:**

A 25-year-old male, presented with complaints of sore throat, nonproductive cough, fever, pleuritic chest pain, and progressive dyspnea for four days. The patient had elevated troponins at presentation of 0.47 (ng/L) that peaked at 4.0 (ng/L). ECG showed sinus rhythm and ST elevations in leads V2, V3, V4, and V5. NT-Pro-BNP was 1740. Transthoracic echocardiogram (TTE) showed reduced ejection fraction (EF) of 37% and global hypokinesis. The rapid strep test was positive for group A streptococcus and C-reactive protein was elevated at 161. Cardiac MRI demonstrated an EF of 53% and edema in the anterior wall without delayed gadolinium enhancement. Cardiac catheterization showed normal coronaries.

**Discussion:**

According to modified Jones criteria, the patient did not meet the full major or minor criteria to be diagnosed with acute rheumatic fever. The course of the nonrheumatic myocarditis is favorable and includes a full recovery of cardiac function, no involvement of cardiac valves, or long-term use of antibiotics.

**Conclusion:**

It is crucial to make a separate distinction between acute rheumatic fever and nonrheumatic myocarditis because this will have huge implications on management and long-term use of antibiotics. Cardiac imaging modalities can aid in distinction between the two disease entities.

## 1. Introduction

Streptococcal pharyngitis is often associated with systemic involvement manifesting as acute rheumatic fever. This can result in damage to all three layers of the heart, the epicardium, myocardium, and endocardium, in a spectrum of disease traditionally known as rheumatic heart disease [1]. Recent reports indicate the presence of an association between streptococcal pharyngitis and myocarditis, which appears to fall outside of the realm of rheumatic fever and rheumatic heart disease [2–6]. This distinct association between streptococcal throat infection and cardiac involvement could broaden our current understanding of cardiac pathology beyond the purview of acute rheumatic fever. We present a case of nonrheumatic streptococcal myocarditis diagnosed with the assist of cardiac MRI.

## 2. Clinical Course

**Figure 1:**
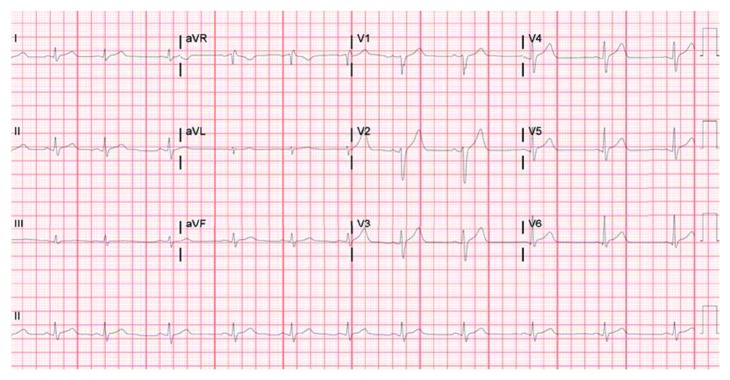
EKG showing normal sinus rhythm with ST elevations in leads V2, V3, V4, and V5.

**Figure 2:**
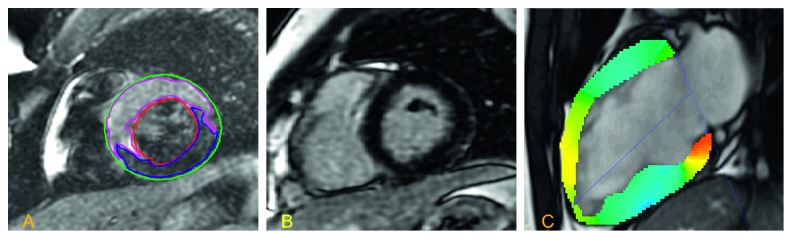
Cardiac MRI showing left ventricular dysfunction with edema and strain abnormalities predominantly in the apical segments suggesting an LAD territory insult. Edema in mid-anterior wall without any late gadolinium enhancement. (a) Increased image intensity of edema-based imaging in the anterior segments. (b) No corresponding late gadolinium enhancement is noted. (c) Movie attached. Still image showing hypokinesis of the anteroapical segments.

A 25-year-old man presented to the hospital with a 4-day history of pleuritic chest pain and progressive dyspnea. He had a sore throat, nonproductive cough, and fever over the same duration of time. His ECG showed sinus rhythm with ST elevations in leads V2, V3, V4, and V5 (Figure 1). Serial troponins showed an upward trend with a peak of 4.0 (ng/L). NT-pro-BNP was 1740 on presentation. Cardiac catheterization showed normal coronary anatomy. A TTE on the same day showed a reduced ejection fraction of 37% with global hypokinesis. The rapid streptococcus antigen test was positive, and CRP was elevated at 161. The patient was presumed to have cardiac manifestations of acute rheumatic fever. Antistreptolysin O (ASO) titers and DNase B strep antibody were low 99.0 IU/ml (0–200) and <78 U/ml (0–120), respectively. Cardiac MRI (CMR) was performed to better visualize and characterize his cardiac pathology. It demonstrated a left-ventricular ejection fraction of 53%. CMR revealed mid-left anterior descending artery territory findings suggestive of edema without evidence of delayed gadolinium enhancement of the myocardium (Figure 2). This finding argued against rheumatic heart disease, which would be expected to involve all three layers of the heart. Isolated involvement of the myocardium in the setting of streptococcal throat infection pointed towards a diagnosis of nonrheumatic streptococcal myocarditis.

## 3. Discussion

Acute rheumatic fever is a result of group A streptococcal pharyngitis, which typically occurs 2 weeks to several months after a throat infection [1]. It is diagnosed using the modified Jones criteria consisting of major, minor, and supportive criteria. The major criteria are carditis (50%–70%), arthritis (35%–66%), chorea (10–30%), subcutaneous nodules (0–10%), and erythema marginatum (<6%). The minor criteria are fever greater than 39°C, prolonged PR interval, elevated C-reactive protein, erythrocyte sedimentation rate, and arthralgia. Supportive criteria for group A *Streptococcus* infection are positive throat culture, rapid streptococcal antigen detection tests, and streptococcal antibody tests [1]. Diagnosis of acute rheumatic fever requires one of the supportive criteria plus two major criteria or one major and two minor criteria. Although our patient's presentation was initially concerning for acute rheumatic fever, he did not meet any major criteria due to isolated myocardial involvement.

Isolated myocarditis after a streptococcal throat infection has been sparsely reported as nonrheumatic streptococcal myocarditis. These reports described young males with cardiac pathology who had full recovery of their cardiac function on follow-up imaging after a course of antibiotics for pharyngitis. Up to our knowledge, five case series were published in literature over the past two decades about nonrheumatic streptococcal myocarditis [2–6]. The average of onset of chest pain was within 7 days of initial throat infection, indicating minimal latency. This indicates a different pathophysiological mechanism of cardiac disease when compared with acute rheumatic fever [1].

Various mechanisms have been proposed to explain myocardial pathology in patients with streptococcal pharyngitis. An early report proposed a possible direct or indirect action of bacterial toxin on the myocardium. A more recent report explored the possibility of molecular mimicry and cross-reactivity between cardiac Ca^2+^ ATPase and group A streptococcal putative calcium-transporting ATPase. Some cases had low titers of ASO in patients with carditis, suggesting a lack of autoimmune phenomenon when compared with rheumatic heart disease [7]. Furthermore, a study suggested that use of antibiotics for bacterial pharyngitis may precipitate nonrheumatic streptococcal myocarditis through bacterial cell wall lysis and release of toxins leading to myocardial damage and necrosis [7].

Cardiac imaging modalities are a vital asset to diagnose the extent and nature of cardiac disease. In our patient, CMR helped demarcate the true extent of cardiac involvement and rule out endocardial involvement, thereby eliminating the possibility of rheumatic heart disease. Most centers use TTE to make this distinction. TTE is valuable to detect subclinical carditis and is increasingly being used to detect disease in the absence of an audible murmur. Furthermore, studies indicate that TEE can be used to differentiate between physiological or trivial murmurs from pathological murmurs of acute rheumatic fever [8]. Therefore, the increasing use of TEE may help appropriately diagnose people with nonrheumatic streptococcal myocarditis in whom acute rheumatic fever was mistakenly assumed due to auscultation of an otherwise benign murmur. This could help prevent unnecessary and prolonged treatment of patients for acute rheumatic fever.

Acute rheumatic fever requires long-term antibiotic prophylaxis to prevent valvular injury [9]. However, in the absence of a clear consensus on the mechanism of cardiac injury, the treatment of nonrheumatic streptococcal myocarditis remains unknown. This is primarily due to an incomplete understanding of the role of antibiotics in the disease process. It has been suggested that antibiotics may shorten the course of disease, worsen the disease due to release of bacterial cell products, or have no bearing on the overall outcome. In the absence of controlled studies without antibiotic treatment, the role of antibiotics remains unclear. Larger studies with control groups are needed to discern the role of antibiotics in patients with nonrheumatic streptococcal myocarditis.

## 4. Conclusion

Acute chest pain in young adults can result from acute rheumatic fever, which can progress to pancarditis involving the valvular apparatus in the absence of appropriate antibiotic treatment. However, it is important to consider that these patients may have chest pain without any valvular disease due to involvement of other structures of the heart. These patients may not fall under the definition of acute rheumatic fever and may not require long-term antibiotic therapy. A growing body of evidence indicates that patients may have nonrheumatic streptococcal myocarditis in the immediate aftermath of streptococcal pharyngitis. In the appropriate context, it remains crucial to test for streptococcal infection while considering the full differential of chest pain in a young patient. Future prospective studies are needed to define the prognosis, pathology, and optimal management of this new disease entity.
